# Effect of osmolytes on *in-vitro* aggregation properties of peptides derived from TGFBIp

**DOI:** 10.1038/s41598-020-60944-0

**Published:** 2020-03-04

**Authors:** Anandalakshmi Venkatraman, Elavazhagan Murugan, Shu Jun Lin, Gary Swee Lim Peh, Lakshminarayanan Rajamani, Jodhbir S. Mehta

**Affiliations:** 1 Singapore Eye Research Institute, 20 College Road, The Academia Building, Singapore, 169856 Singapore; 2 Ophthalmology and Visual Sciences Academic Clinical Program, Duke-NUS Graduate Medical School, Singapore, 169857 Singapore; 3 Singapore National Eye Centre, 11 Third Hospital Avenue, Singapore, 168751 Singapore

**Keywords:** Biophysics, Medical research

## Abstract

Protein aggregation has been one of the leading triggers of various disease conditions, such as Alzheimer’s, Parkinson’s and other amyloidosis. *TGFBI*-associated corneal dystrophies are protein aggregation disorders in which the mutant TGFBIp aggregates and accumulates in the cornea, leading to a reduction in visual acuity and blindness in severe cases. Currently, the only therapy available is invasive and there is a known recurrence after surgery. In this study, we tested the inhibitory and amyloid dissociation properties of four osmolytes in an *in-vitro* *TGFBI* peptide aggregation model. The 23-amino acid long peptide (TGFBIp 611–633 with the mutation c.623 G>R) from the 4th FAS-1 domain of TGFBIp that rapidly forms amyloid fibrils was used in the study. Several biophysical methods like Thioflavin T (ThT) fluorescence, Circular Dichroism (CD), fluorescence microscopy and Transmission electron microscopy (TEM) were used to study the inhibitory and amyloid disaggregation properties of the four osmolytes (Betaine, Raffinose, Sarcosine, and Taurine). The osmolytes were effective in both inhibiting and disaggregating the amyloid fibrils derived from TGFBIp 611–633 c.623 G>R peptide. The osmolytes did not have an adverse toxic effect on cultured human corneal fibroblast cells and could potentially be a useful therapeutic strategy for patients with TGFBIp corneal dystrophies.

## Introduction

Intracellular and extracellular aggregation of proteins is often mediated by regions of low compositional complexity that may be intrinsically disordered in the native proteins^1,2^. The irreversible pathological deposition of protein aggregates has been associated with diseases including Alzheimer’s, Parkinson’s and prion diseases^3–5^. Stromal corneal dystrophies associated with mutations in the Transforming Growth Factor-Beta Induced (*TGFBI*) gene, located on chromosome 5q31^5–8^ are inherited protein aggregation disorders. They are characterized by age-dependent gradual accumulation and deposition of protein aggregates in various depths of the stroma leading to loss of corneal transparency and vision impairment^5^. The protein product of the *TGFBI* gene is a secreted 68-kDa extracellular matrix protein found in tissues throughout the body yet only the cornea is affected by the mutant proteins^9,10^.

TGFBIp consists of a secretory signal peptide, a domain rich in cysteine residues, a cell attachment tripeptide Arg-Gly-Asp (RGD) signal residue, and four Fasciclin 1 (FAS-1) domains^9,10^. The *TGFBI* gene has 74 mutations reported to date^11^, and the mutant protein is associated with modified protein stability, altered proteolytic processing, and deposition of insoluble aggregates in various layers of the cornea^12–16^. The aggregation and deposition of TGFBIp display different clinical phenotypes; the deposits range from amyloidogenic structures to amorphous granular deposits, or a combination of both^17^. Mutations in TGFBIp are not only known to alter the turnover rate but also alter the thermodynamic stability of the protein with several of the mutations leading to destabilized protein which is more likely to unfold^18–20^. The mutant protein also possesses different proteolytic processing and clearance mechanism in the eye compared to the wild type protein (WT). These proteolytic products can act as amyloid seeds that could trigger the TGFBIp aggregation pathway.

It has been reported that the peptides derived from the 1^st^ and 4^th^ FAS-1 domains of mutant TGFBIp have increased aggregation propensity compared to the WT^21–24^. We have also previously reported the protein composition of the amyloid fibrils from Lattice Corneal Dystrophy patients (LCD) using mass spectrometric assays^25^. The proteolytic fragments of TGFBIp derived from amyloid fibrils of patients, upon digestion with trypsin showed a higher abundance of peptides from the 4^th^ FAS-1 domain of TGFBIp compared to healthy controls. Peptides G^511^DNRFSMLVAAIQSAGLTETLNR^533^, Y^571^HIGDEILVSGGIGALVR^588^, E^611^PVAEPDIMATNGVVHVITNVLQPPANRPQER^642^ and L^497^TPPMGTVMDVLKGDNRFSML

VAAIQSAGLTETLNR^533^ were enriched in the patient samples compared to the controls^26^. The peptide region E^611^PVAEPDIMATNGVVHVITNVLQ^633^ ^26^ has been associated with more than 16 clinically significant mutations, with high potential to form amyloid fibrils even under physiological conditions^24,27^. Eleven mutations in this peptide region are known to alter the overall net charge of TGFBIp^26^. Based on the *in-vitro* aggregation properties of peptides, TGFBIp ^611–633^ c.623 G>R and TGFBIp ^611–633^ c.622 N>K exhibited greater propensity to form amyloid fibrils and displayed remarkable resistance to thermal denaturation when compared to WT fibrils. The *TGFBI* c.623 G>R was first clinically reported by Gruenauer-Kloevekorn *et al*. in 2009 in two German families^28^.

Osmolytes are small organic molecules, of diverse chemical structures, that regulate the solvent properties of cells, by conserving the native structures of proteins during an osmotic or thermal stress response^29–32^. Osmolytes may be categorized as polyhydric alcohols, sugars (polyols), amino acids (and their derivatives), and methylammonium compounds^32–35^. Osmolytes are widely used to stabilize and facilitate protein folding since they can act as “chemical chaperones”^36,37^. Osmo-protectants and chemical chaperones have been shown to shift the equilibrium towards the native state^33^, by exhibiting a thermodynamic stabilization of the protein. This is accomplished by repopulating the denatured and native states, via unfavorable interactions with protein surfaces (a combination of backbone and side-chain interactions). Hence recently, more osmolytes are being evaluated as modes of treatment in various protein aggregation related disorders like Alzheimer’s disease and Huntington’s disease^38,39^.

The purpose of this study was to investigate the role of osmolytes on the aggregation properties of model peptide TGFBIp ^611–633^ with the mutation c.623 G>R (referred to as TGFBIp ^611–633^ G623R peptide in this manuscript). We chose four osmolytes Raffinose^40^, Taurine^35,41,42^, Betaine^35,42,43^ and Sarcosine^35,42,43^ (Supplementary Fig. 1) that have been well known to aid in protein folding but also prevent protein aggregation. The osmolytes contain numerous hydrogen bonding donors/acceptors which may interfere with the β-amyloid oligomers or fibrils. We employed various biophysical, biochemical and microscopic methods such as Thioflavin T (ThT) fluorescence assay, circular dichroism spectroscopy and electron microscopy to investigate the inhibition and disaggregation effect of osmolytes on the amyloidogenicity of TGFBIp ^611–633^ G623R peptide. We then investigated the cytotoxicity of the osmolytes on human corneal fibroblasts (HCF).

## Results

Live-cell imaging (Supplementary Fig. 2) of HCFs when exposed to various concentrations of osmolytes (0.1 mM, 1 mM, 10 mM, 100 mM and 1000 mM) indicated no apparent changes in the cell numbers and cell density. The cells proliferated in the presence of osmolytes indicating that the osmolytes did not have an inhibitory effect on the proliferation of HCF.

**Figure 1:**
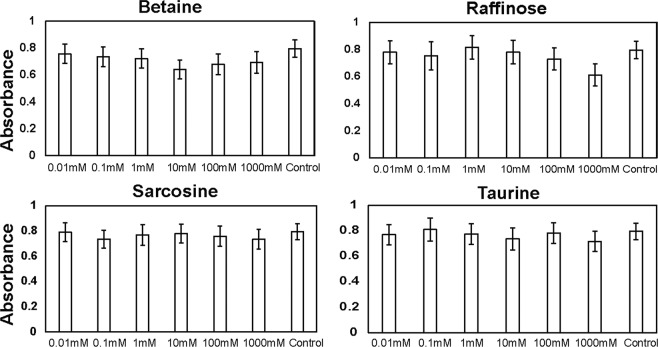
Cytotoxicity of osmolytes determined by MTT assays. All four osmolytes (Betaine, Raffinose, Sarcosine and Taurine) even at higher concentrations did not show any significant cytotoxic properties.

The effect of various concentrations of osmolytes on HCF when assessed by 3-(4, 5-Dimethylthiazol-2-yl)-2, 5-Diphenyltetrazolium Bromide (MTT) (Fig. 1) assays did not show any significant cytotoxic properties. Statistical analysis performed on the observed absorbance values of different concentration of osmolytes compared to PBS treated controls did not show any statistical significance between the osmolytes treated versus the controls. These results suggest that the four osmolytes even at the elevated concentrations (1 M) are non-cytotoxic to the HCF.

### Effect of osmolytes on amyloid fibril formation

**Figure 2:**
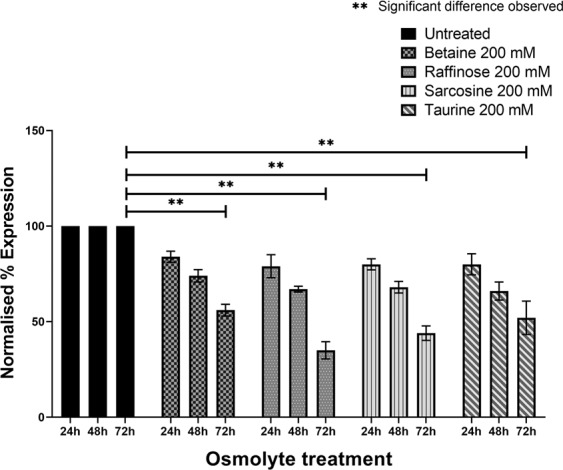
Inhibition of amyloid fibrillation of TGFBIp 611–633 G623R peptide by osmolytes determined by ThT fluorescence assays. ThT fluorescence assay indicated a time-dependent decrease in the ThT fluorescent intensity for the peptides treated with the four osmolytes when compared to the control-treated with PBS.

**Figure 3:**
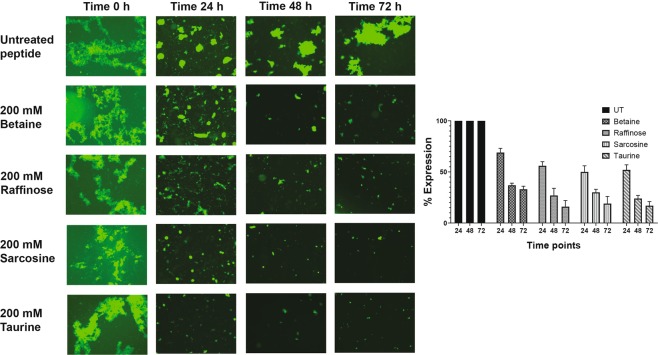
Inhibition of amyloid fibrillation determined by ThT fluorescent microscopy images of the TGFBIp 611–633 G623R peptides treated with betaine, raffinose, sarcosine and taurine, respectively, at various time points. Fluorescence images of the peptide samples treated with the osmolytes show smaller and fewer fluorescent spots compared to the control-treated with PBS. The fluorescence images of mutant peptide samples treated with taurine showed relatively smaller fluorescent spots as early as 24 h compared to the samples treated with other osmolytes.

The kinetics of fibril formation, monitored by ThT dye at 24, 48 and 72 h post-incubation indicated a time-dependent decrease in the ThT fluorescent intensity for the peptides incubated with various osmolytes when compared to PBS treatment (Fig. 2). The effect was discernible even from 24 h post-incubation. At around 72 h the inhibitory effect was more pronounced for raffinose (65% ± 5), sarcosine (56% ± 4) (Table 1) followed by taurine (48% ± 9) and betaine (44% ± 3). The results demonstrate that all the four osmolytes inhibited amyloid fibril formation of *TGFBI-*peptide. The inhibitory effects of osmolytes were investigated further by fluorescence microscopy after staining with ThT (Fig. 3). In agreement with the ThT assay, fluorescence images demonstrated the strong inhibitory effects of osmolytes on the amyloid fibril formation of TGFBIp ^611–633^ G623R peptide. All osmolytes displayed inhibition of amyloid fibril formation of the peptide around 72 h. The effect of taurine on the amyloid fibril formation showed relatively smaller fluorescent spots even after 24 h compared to the peptide treated with other osmolytes.

**Table 1:** (a) Inhibition of amyloid fibrillation of TGFBIp ^611–633^ G623R peptide by osmolytes determined by ThT fluorescence assay. (b) Comparison of θ[218] values of untreated and osmolyte-treated peptides at various time points determined by circular dichroism assays.

Osmolyte	Treatment Time	% Inhibition (mean ± SD) n = 3
**(a)**		
Untreated (PBS)	24 h	0
	48 h	0
	72 h	0
Betaine	24 h	16 ± 3
	48 h	26 ± 3
	72 h	44 ± 3
Raffinose	24 h	21 ± 6
	48 h	32 ± 2
	72 h	65 ± 5
Sarcosine	24 h	20 ± 3
	48 h	32 ± 2
	72 h	56 ± 4
Taurine	24 h	20 ± 6
	48 h	34 ± 5
	72 h	48 ± 9
**(b)**		
Betaine	24 h	8 ± 1
	48 h	19 ± 6
	72 h	34 ± 10
Raffinose	24 h	19 ± 8
	48 h	23 ± 10
	72 h	57 ± 10
Sarcosine	24 h	17 ± 6
	48 h	43 ± 10
	72 h	57 ± 8
Taurine	24 h	25 ± 4
	48 h	54 ± 9
	72 h	56 ± 12

**Figure 4:**
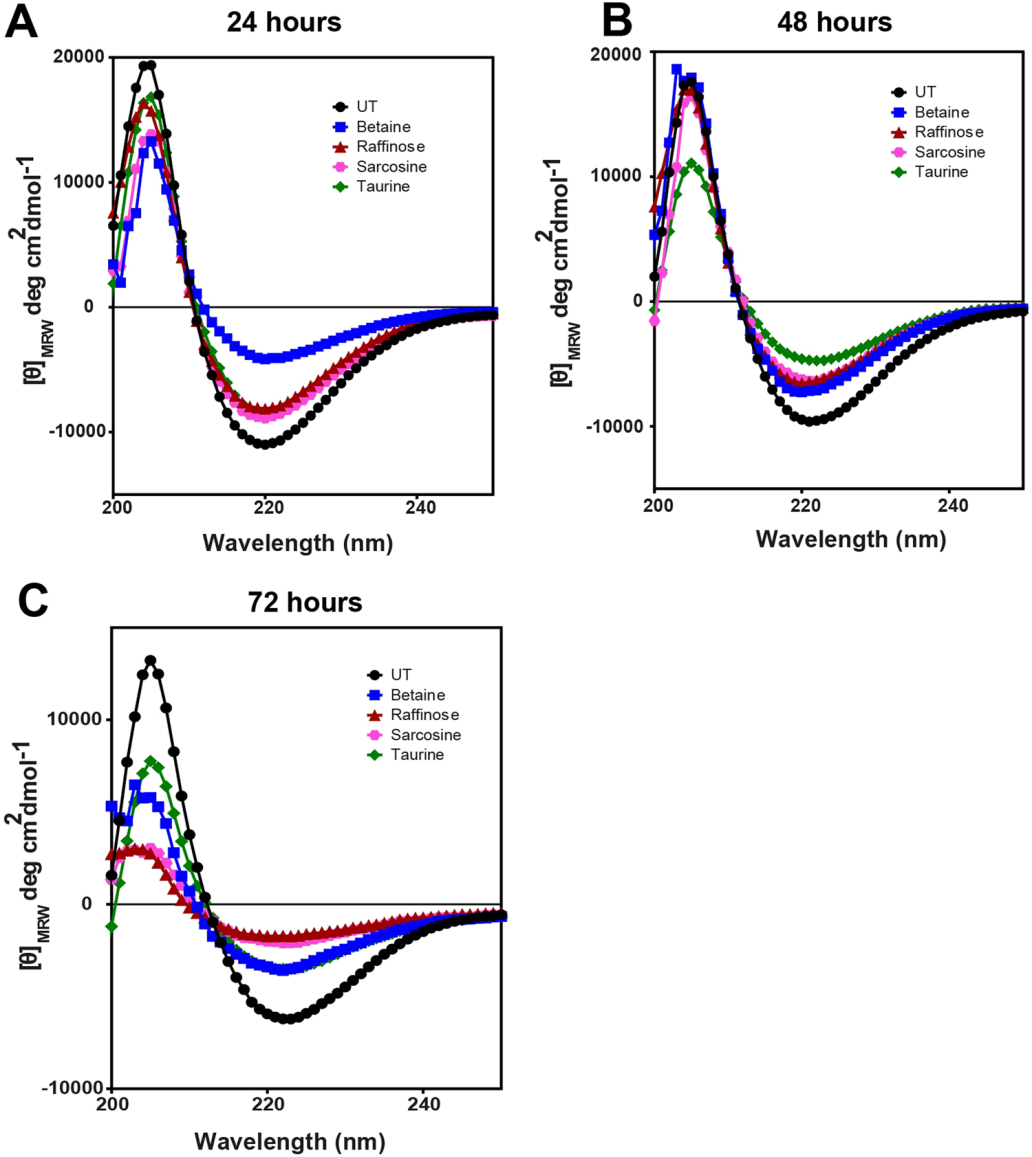
(**A–C**). Inhibition of amyloid fibrillation determined by Circular Dichroism assays of the untreated TGFBIp 611–633 G623R peptide and the peptide samples treated with various osmolytes and followed at 24 h, 48 h, and 72 h respectively.

Changes in the secondary structure of the peptide in the presence of osmolytes were followed by Circular Dichroism assays. The far UV-CD data without any osmolyte treatment after 24 h of fibrillation shows clear negative minima around 218 and a positive peak around 195 which are characteristics of β-sheet secondary structure (Fig. 4) confirming high aggregation propensity of the native peptide to a form ordered structures within 24 h.

For peptide incubated with 200 mM Betaine, (Fig. 4) the ellipticity of peaks around 218 and 195 decreased progressively. The comparison of θ _[218]_ value between the native peptide and 200 mM betaine treated showed 8% ± 1, 19% ± 6 and 34% ± 10 reduction (Table 1) in the peak intensity at 24, 48 and 72 h respectively compared to the untreated sample.

For peptide incubated with raffinose, a decreased intensity minima around 218 and a positive peak around 195 was still observed (Fig. 4). The θ _[218]_ values show that the fibrillation of native peptide was inhibited by 19% ± 8, 23% ± 10 and 57% ± 10 at the 3 different time points respectively. Raffinose and Sarcosine showed maximum inhibition of TGFBIp ^611–633^ c.623 G>R peptide (Fig. 4). Visible characteristics of β-sheet amyloid fibrils were observed after 24 h and θ _[218]_ value observed at three different time points showed about 17% ± 6, 43% ± 10 and 57% ± 8 inhibition of fibril formation when treated with sarcosine. With 200 mM taurine, the inhibition of fibrillation was observed with a reduction in characteristic β-sheet secondary structure and about 25% ± 4, 54% ± 9 and 56% ± 12 inhibition of fibrillation at the time points (Fig. 4).

### Role of osmolytes in the disaggregation of amyloid fibrils

**Figure 5:**
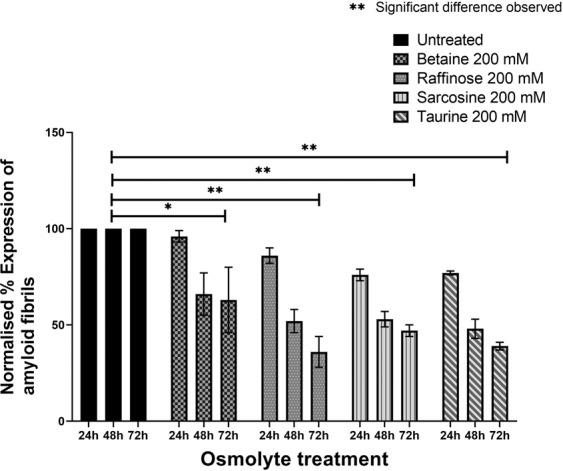
Disaggregation of amyloid fibrillation of TGFBIp 611–633 G623R peptide by osmolytes determined by ThT fluorescence assays. Treatment of the preformed amyloid fibrils with osmolytes resulted in a time-dependent decrease in the ThT fluorescent intensities. Among the four osmolytes, raffinose and taurine treatment resulted in a significant decrease in ThT intensity by 64% ± 8 (raffinose) and 61% ± 2 (taurine), respectively, at 72 hours after treatment.

The preformed amyloid fibrils were treated with an optimal working concentration (200 mM) of osmolytes (raffinose, betaine, sarcosine, and taurine) for 24, 48 and 72 h and their ability to disaggregate the preformed amyloid fibrils were assessed as before (Fig. 5). Treatment of the matured fibrils with osmolytes resulted in a time-dependent decrease in the ThT fluorescent intensities. Among the four osmolytes, raffinose (64% ± 8) and taurine (61% ± 2) (Table 2) treatment resulted in a significant decrease in ThT intensity after 72 h, suggesting that the osmolytes could disrupt the non-covalent interactions of the β-sheet assembly.

**Table 2:** (a) Dissolution of amyloid fibrils from TGFBIp ^611–633^ G623R peptide by osmolytes studied with ThT fluorescence assay. (b) Comparison of θ[218] values of untreated and osmolyte-treated preformed amyloid fibrils.

Osmolyte	Treatment Time	% Inhibition (mean ± SD) n = 3
**(a)**		
Untreated (PBS)	24 h	0
	48 h	0
	72 h	0
Betaine	24 h	4 ± 3
	48 h	34 ± 11
	72 h	37 ± 17
Raffinose	24 h	14 ± 4
	48 h	48 ± 6
	72 h	64 ± 8
Sarcosine	24 h	24 ± 3
	48 h	47 ± 4
	72 h	53 ± 3
Taurine	24 h	23 ± 1
	48 h	52 ± 5
	72 h	61 ± 2
**(b)**		
Betaine	24 h	6 ± 1
	48 h	24 ± 11
	72 h	36 ± 5
Raffinose	24 h	7 ± 7
	48 h	48 ± 9
	72 h	58 ± 11
Sarcosine	24 h	12 ± 8
	48 h	39 ± 6
	72 h	41 ± 2
Taurine	24 h	13 ± 6
	48 h	40 ± 12
	72 h	54 ± 6

**Figure 6:**
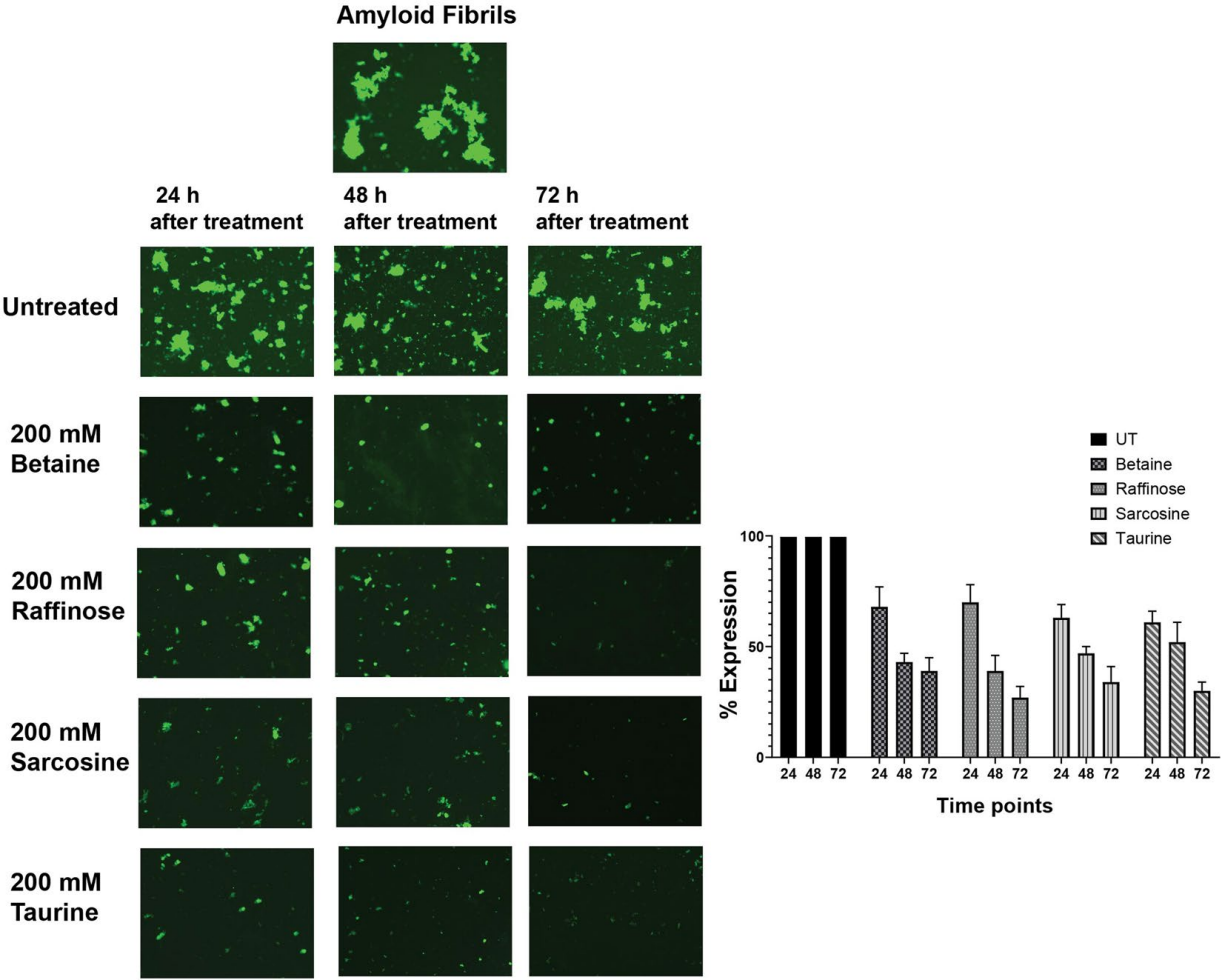
Disaggregation of amyloid fibrillation determined by ThT fluorescent microscopy images of the TGFBIp ^611–633^ G623R peptides treated with betaine, raffinose, sarcosine and taurine, respectively, at various time points. Fluorescence images indicate that a marked decrease in fluorescence staining for all the four osmolytes after 24 h post-treatment.

The preformed amyloid fibrils from the peptide were treated with osmolytes and followed up to 72 h post-treatment and visualized under the fluorescence microscope by adding ThT dye (Fig. 6). The results indicated a marked decrease in fluorescence staining for all the osmolytes after 24 h post-treatment. A progressive decrease in ThT staining was observed for all the osmolytes treated samples after 48 and 72 h post-treatment, suggesting disaggregation of the preformed amyloid fibrils. This clearly shows that all osmolytes effectively disaggregate preformed amyloid fibrils from TGFBIp ^611–633^ G623R peptide.

**Figure 7:**
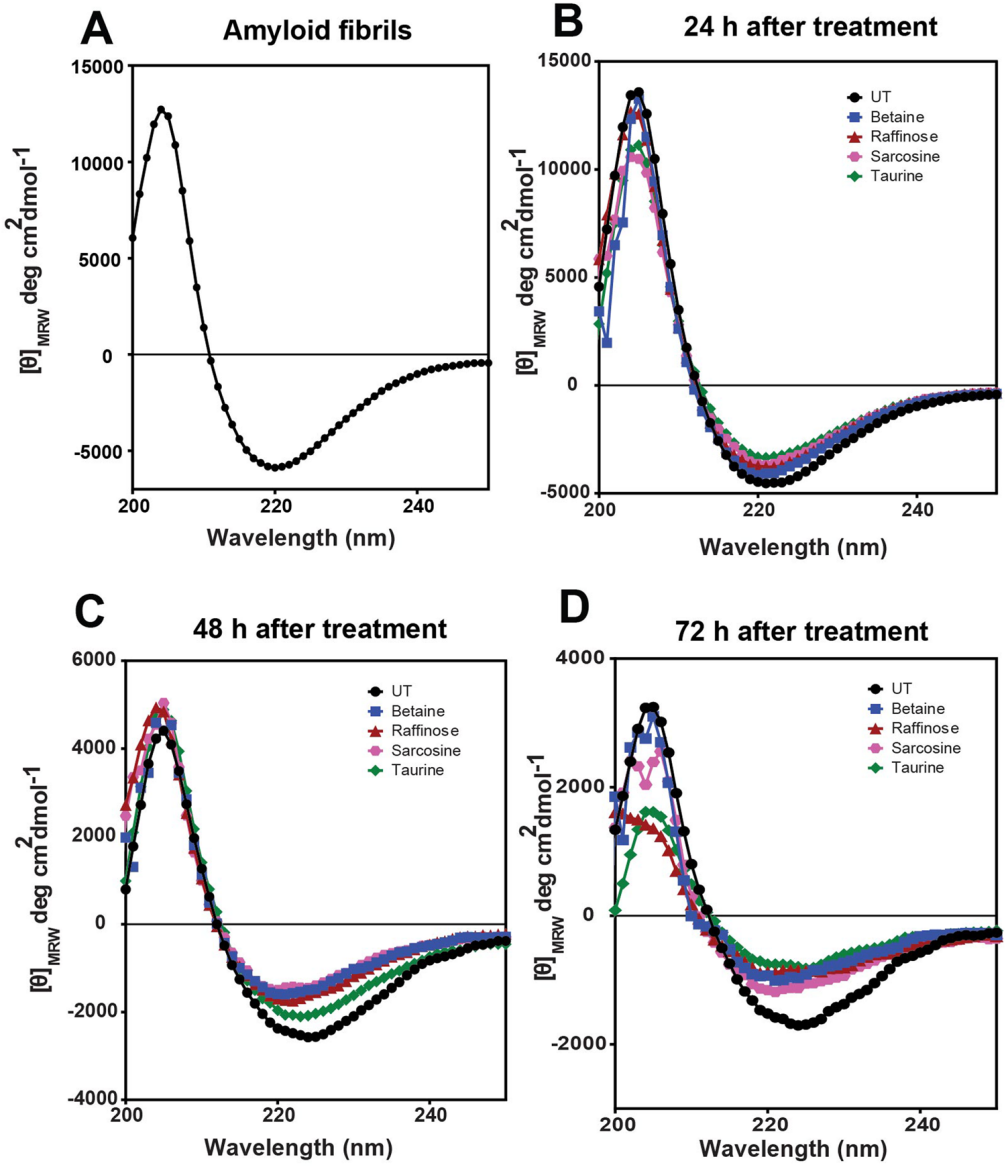
(**A–D**). Disaggregation of amyloid fibrillation determined by Circular Dichroism assays of the untreated TGFBIp 611–633 G623R peptide and the peptide samples treated with various osmolytes. (**A**) shows the formation of amyloid fibrils with the typical β-sheet structure. (**B–D**) shows the disaggregation by osmolytes followed after 24 h, 48 h and 72 h after osmolyte treatment respectively. CD assay showed a marked decrease in the amplitude of the peak at [θ]_218_, which suggests disruption and disaggregation of the preformed amyloid fibrils. Among the four osmolytes, raffinose and taurine displayed the highest disruption of the β-sheet structure of the amyloid fibrils compared to betaine and sarcosine.

The disaggregation effect studied by far UV-CD experiments of native TGFBIp ^611–633^ G623R peptide showed absorption minima around 218 nm and absorption maxima around 195 nm (Fig. 7, Table 2) which is very typical for a β-sheet rich secondary structure of amyloid fibrils. This shows that the peptide formed amyloid fibrils within 24 h, the presence of amyloid fibrils was confirmed by TEM analysis. Amyloid fibrils treated with osmolytes and followed after 72 h showed a marked decrease in the amplitude of the peak at [θ]_218_. Among the different osmolytes Raffinose and Taurine showed around 58% ± 11 and 54% ± 6 dissolution respectively followed by sarcosine (41% ± 2) and betaine (36% ± 5) suggesting disruption of the preformed amyloid fibrils, and corroborate with the ThT results. Among the osmolytes, raffinose and taurine displayed the highest disruption of the b-sheet structure when compared to betaine and sarcosine (Fig. 7).

**Figure 8:**
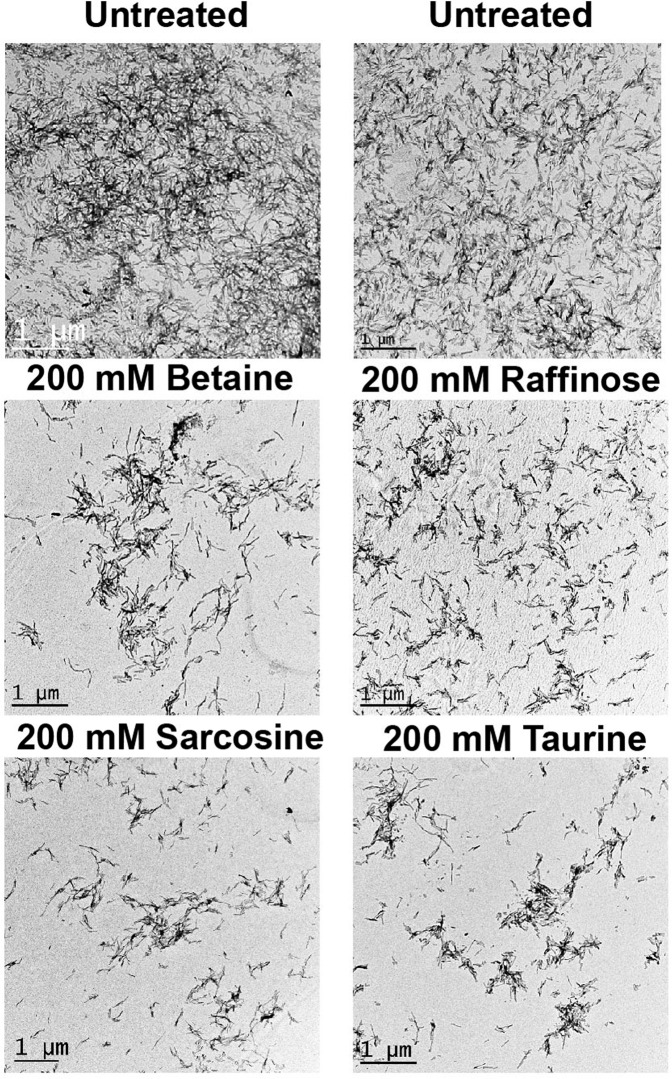
Transmission Electron Microscopy (TEM) images of the preformed amyloid fibrils from TGFBIp ^611–633^ G623R peptide treated with betaine, raffinose, sarcosine and taurine for 72 h, respectively. When treated with osmolytes, the TEM image showed disaggregation of amyloid fibrils with the absence of densely packed amyloid fibrils.

The disaggregation of preformed amyloid fibrils was also verified by TEM analysis (Fig. 8). The long bundle like morphology, typical of amyloid fibrils was visible for the untreated native TGFBIp ^611–633^ G623R peptide. Addition of osmolytes to the amyloid aggregates showed disaggregation of amyloid fibrils with the absence of densely packed amyloid fibrils. The reduction in density of ordered amyloid fibrils was evident when the amyloid fibrils were treated with raffinose, betaine, sarcosine and taurine. The results are also in agreement with the ThT fluorescence, circular dichroism and immunofluorescence imaging.

## Discussion

The formation of insoluble amyloid aggregates is a pathological hallmark of *TGFBI*-associated corneal dystrophies. In our recent work, we compared the biological properties of a 23-residue peptide fragment that was highly abundant in the patient’s cornea who carried the c.626 H>R mutation. Decreasing the overall net charge of the 23-residue peptide (amino acid 611 to 633) by substitution with cationic residues resulted in a position-dependent alteration in the kinetics of amyloid formation^26^. Among these peptides, amyloid fibrils formed by TGFBIp ^611–633^ G623R peptide contained homogenous populations of β-sheet assemblies, displayed excellent thermal stability when compared to other peptides. Thus, we used TGFBIp ^611–633^ G623R as a model peptide for assessing the efficacy of various osmolytes on amyloid inhibition as well as disaggregation efficiency.

All the investigated osmolytes were non-cytotoxic to HCFs even at a final concentration of 1 M in the cell culture media. When incubated alone, the peptide TGFBIp ^611–633^ G623R rapidly aggregated to form amyloid aggregates in 24 h whereas incubation of peptides with an equimolar concentration of osmolytes resulted in a time-dependent decrease in amyloid formation. Preformed amyloid fibrils treated with osmolytes and monitored by ThT intensity, CD spectropolarimetry, fluorescence imaging, and TEM indicated Raffinose and taurine displayed significant dissolution of the amyloid fibrils. All observations confirm the effectiveness of osmolytes in the inhibition of amyloid fibril formation and the dissolution of amyloid fibrils. Among the four osmolytes tested raffinose and taurine have excellent amyloid inhibition as well as disaggregation properties.

There have been few previously reported studies on TGFBIp model peptides and the effect of chemical compounds on amyloid fibril formation and treatment options to disaggregate amyloid fibrils. Kato *et al*. reported that the presence of preservatives such as benzalkonium chloride in eye drops close to the critical micellar concentration (0.001–0.02% (0.03–0.6 mM) accelerated the amyloid fibril formation in TGFBIp derived peptides and highlighted the potential risks associated with such formulations^44^ in eye drops. Use of several organic, polymeric, and inorganic nanoparticles, synthetic peptides, amino acids and sialic acid^45–47^ were reported to inhibit amyloid fibril formation. Palmal and Debnath *et al*., reported the utility of curcumin conjugated gold nanoparticles and epigallactocatechin-3-gallate conjugated polymer nanoparticles for inhibition and dissolution of preformed amyloid fibrils of Aβ_1–40_ peptide^48,49^. The strong affinity of nanoparticles for both oligomeric and fibrillar form of peptides was suggested to be responsible for stabilization of soluble oligomers which in turn attenuated the neurotoxicity of β-oligomers^48,49^. Unlike β-oligomers formed by Aβ_1–40_ peptide, the soluble oligomers of TGFBIp 611–633 c.623 G>R were non-toxic^26^. Thus, the dissolution of amyloid fibrils from TGFBIp by osmolytes may not have an adverse effect on the tissue and could potentially be a useful therapeutic strategy for patients with corneal dystrophies.

Using a 22 residue model peptide derived from 1^st^ FAS1 domain of *TGFBI*, Ozawa *et al*., have reported the use of a helium-cadmium laser with a power of 60–80 milliwatts at 37 °C under continuous agitation for disintegrating of amyloid fibrils^50^. Clinically, LASIK treatment in patients with corneal dystrophies worsens corneal haze and accelerated disease progression^51,52^. Byrne and Angell used protic ionic liquids for the disintegration of amyloid fibrils formed by hen egg-white lysozyme and the dissolved lysozyme retained substantial enzymatic activity^53^. A similar mechanism may hold good for the dissolution of TGFBIp amyloid fibrils by osmolytes. To the best of our knowledge, this is the first report on the use of non-cytotoxic osmolytes for the inhibition and disintegration of amyloid fibrils derived from *TGFBI* associated corneal dystrophies.

There have been several previous attempts to generate a suitable transgenic animal model, either to knock-in or knock-out *TGFBI* gene, and to evaluate the pathologic role of the mutant protein^54–56^. All the generated animal models were not very successful to express the disease phenotype or in the survival of the animals. Since there are more than 65 mutations reported in this disease, it will not be feasible to generate animal models that represent each mutation or a universal model that can be used to study all the mutant phenotypes. Hence, the *in-vitro* peptide aggregation model will be more useful to study any treatment options that can be used to either prevent protein aggregation or dissolve preformed aggregates.

## Materials and Methods

### Materials

A synthetic peptide (TGFBIp ^611–633^ c.623 G>R) with the amino acid change (Glycine to Arginine) was purchased from Synpeptide Co Ltd, Shanghai, China. The peptides were 95% homogenous as confirmed by reversed-phase high-performance liquid chromatography (Supplementary Fig. 3). Thioflavin T (ThT) and the osmolytes were purchased from Sigma-Aldrich (Sigma-Aldrich Inc., MO). Formvar-carbon coated nickel grids were bought from EMS (Electron Microscopy Sciences, PA).

### Cytotoxicity of osmolytes on cultured human corneal fibroblast (HCF)

#### Real-time live-cell imaging by IncuCyte

For real-time observation of the effect of the osmolytes on cultured human corneal fibroblast, 3000 cells/well were seeded in 96-well plates and placed in an incubator at 37 °C for 24 h to proliferate. Cells were then incubated with varying concentrations of the osmolytes (0.1 mM, 1 mM, 10 mM, 100 mM and 1000 mM) in triplicates and images were captured with IncuCyte ZOOM System (Essen BioScience Inc., Research Instruments, Singapore). Frames were then captured at 4-h intervals from 4 separate regions/well using a 10× objective for 20 h to observe cell toxicity.

### Cell viability by MTT assay

Cell viability of cultured human corneal fibroblast upon addition of osmolytes was assessed with Vybrant MTT Cell Proliferation Assay Kit (Life Technologies, #V13154). Culture media was replaced with 100 µl of PBS followed by the addition of 10 µl of 12 mM MTT solution. Cells were incubated in the dark at 37 °C for 3 hours before dissolving of formazan crystals with DMSO through the removal of all but 25 µl of medium and adding 50 µl of DMSO followed by thorough mixing and incubation at 37 °C for 10 minutes as per the manufacturer’s protocol. Absorbance was taken at 540 nm with a microplate reader (Tecan Infinite 200 PRO).

### *In-vitro* amyloid fibrillation

The 23-amino acid long peptide (TGFBIp ^611–633^ c.623 G>R) from the 4^th^ FAS1 domain of TGFBIp with the substitution, G623→R(EPVAEPDIMATNRVVHVITNVLQ) that rapidly formed amyloid fibrils was used in this study. The peptide was dissolved (0.6 mg/ml) in PBS and allowed to form amyloid fibrils in 50 ml Falcon tubes in a shaking incubator at 37 °C and 180 rpm with and without the addition of osmolytes.

### Thioflavin T (ThT) assay

To study the Inhibitory effect of osmolytes in amyloid fibril formation, samples were allowed to fibrillate in 50 ml Falcon tubes with the osmolytes (Betaine, Raffinose, Sarcosine, and Taurine). The working concentration of all the osmolytes were 200 mM. The molecular weight of the various osmolytes used in the study is given in Supplementary Table 1. The peptide solution at various time points were treated with 30 µM thioflavin T (ThT) in PBS buffer at pH 5.5 in a Greiner 96-well flat-bottom polystyrol microplate (Greiner, Frickenhausen Germany), and the resulting fluorescence was recorded using a microplate reader. The reaction was followed for 24, 48, 72 hours. To examine the disaggregation properties of osmolytes, ThT experiments were also performed 24, 48 and 72 hours after the samples were allowed to form amyloid fibrils for 24 hours. The samples were excited at 445 nm and the resulting emission fluorescence at 485 nm was measured using a microplate reader (Tecan infinite M200 pro, Zanker Road, San Jose, USA). Percentage inhibition for each osmolyte treatment was calculated by subtracting the baseline fluorescent intensity without the peptides and compounds from the observed fluorescent intensity of each treatment well. The fluorescent intensities per osmolyte treatment were normalized against the untreated fluorescent intensity per time point and expressed as a percentage.

### Circular dichroism spectroscopy

Far UV–CD spectra of the TGFBIp ^611–633^ c.623 G>R peptide aggregation in the presence of various osmolytes at 24, 48 and 72 hours were measured. Dissolution of amyloid fibrils incubated with various osmolytes for 24, 48 and 72 hours after 24 hours of TGFBIp ^611–633^ G623R peptide fibrillation process in PBS buffer at pH 7.0 was collected in a Chirascan plus spectropolarimeter (Chirascan, Applied Photophysics, UK) using a 0.1 cm path length quartz cuvette. Spectra were recorded from 260 nm to 190 nm in 0.1 nm steps at a scan rate of 50 nm/min. The final spectrum was the average of three scans as per the manufacturer’s recommendation. The Mean Residual Weight (MRW) ellipticity ([θ]_mrw_) at wavelength λ was calculated using the equation:

$${[{\rm{\theta }}]}_{{\rm{mrw}}}={\rm{MRW}}\times {{\rm{\theta }}}_{\lambda }/10\times {\rm{l}}\times {\rm{c}}$$

where θ_λ_ is the observed ellipticity at a particular wavelength (degrees), l is the path length (cm), and c is the concentration (g/ml). Secondary structures of the protein were analyzed using CDNN software.

### Calculation: [θ] 218

The percentage inhibition of secondary beta-pleated structure for inhibition and dissolution of fibrils of the peptide in the presence of osmolytes was calculated using [θ] _218_ values. Percentage inhibition for each osmolyte treatment per time point was calculated by normalizing [θ] _218_ of osmolyte treatment with the [θ] _218_ of untreated wells and expressed as a percentage.

### Fluorescent microscopy

For both inhibition of TGFBIp ^611–633^ c.623 G>R peptide amyloid fibril formation by osmolytes and dissolving the preformed amyloid fibrils, the samples were incubated with 1:1 ratio of the peptide solution to ThT in the dark for 30 minutes. 25 µl of the samples were taken on to slides with coverslips and visualized under a fluorescent microscope (AxioImager Z1, Carl Zeiss, Oberkochen, Germany). Three representative images from each time point under a given condition was used for the quantitation of fluorescence. Image J software was used to quantify the signal from the images and the values for each time point per osmolyte treatment were normalised to the untreated sample at that particular time point.

### Transmission electron microscopy (TEM)

Morphological analysis of the TGFBIp ^611–633^ c.623 G>R fibrils before and after treatment with osmolytes for 72 hours was investigated by Transmission electron microscopy (TEM) with a JEOL JEM-1010 transmission electron microscope using Digital Micrograph ™ 1.81.78 for GMS 1.8.0 (Gatan, Pleasanton, CA) at the Singhealth-core Electron Microscopy facility. 10 µl of amyloid fibril samples with and without osmolyte treatment were applied onto Formvar-carbon coated 300-mesh-size nickel grids. The samples were stained with Uranyl acetate solution, washed, dried, and observed at magnifications 8000–50000X at 80 kV.

### Statistical analysis

All numeric data obtained were expressed as mean ± standard deviation. For cytotoxicity studies, all the comparisons were done using one-way ANOVA followed by post-hoc Bonferroni test (SPSS Statistics 22.0, IBM Chicago, IL, USA) for multiple comparisons. For both the experiments, values were deemed to be significant (*) when a *p*-value of less than 0.05 was achieved and very significant (**) with a *p*-value of less than 0.01 was achieved^57^.

## Supplementary information

Supplementary information.
