# Supplementary material for: Effect of osmolytes on in-vitro aggregation properties of peptides derived from TGFBIp
Source: Sci Rep. 2020 Mar 4;10:4011. doi: 10.1038/s41598-020-60944-0 (PMC7055237; doi:10.1038/s41598-020-60944-0)

Supplementary information

Effect of osmolytes on in-vitro aggregation properties of peptides derived from TGFBIp

Venkatraman Anandalakshmi [1], Elavazhagan Murugan [1,2], Lin Shu Jun[1], Gary Swee Lim Peh[1, 2], R. Lakshminarayanan [1,2#], Jodhbir S. Mehta [1,2,3 #]

[1]Singapore Eye Research Institute, 11 Third Hospital Avenue, Singapore 168751
[2]Ophthalmology and Visual Sciences Academic Clinical Program, Duke-NUS Graduate Medical School, Singapore, Singapore 169857
[3]Singapore National Eye Centre, 11 Third Hospital Avenue, Singapore 168751

Supplementary Figure 1

Structure of Osmolytes used in the study.

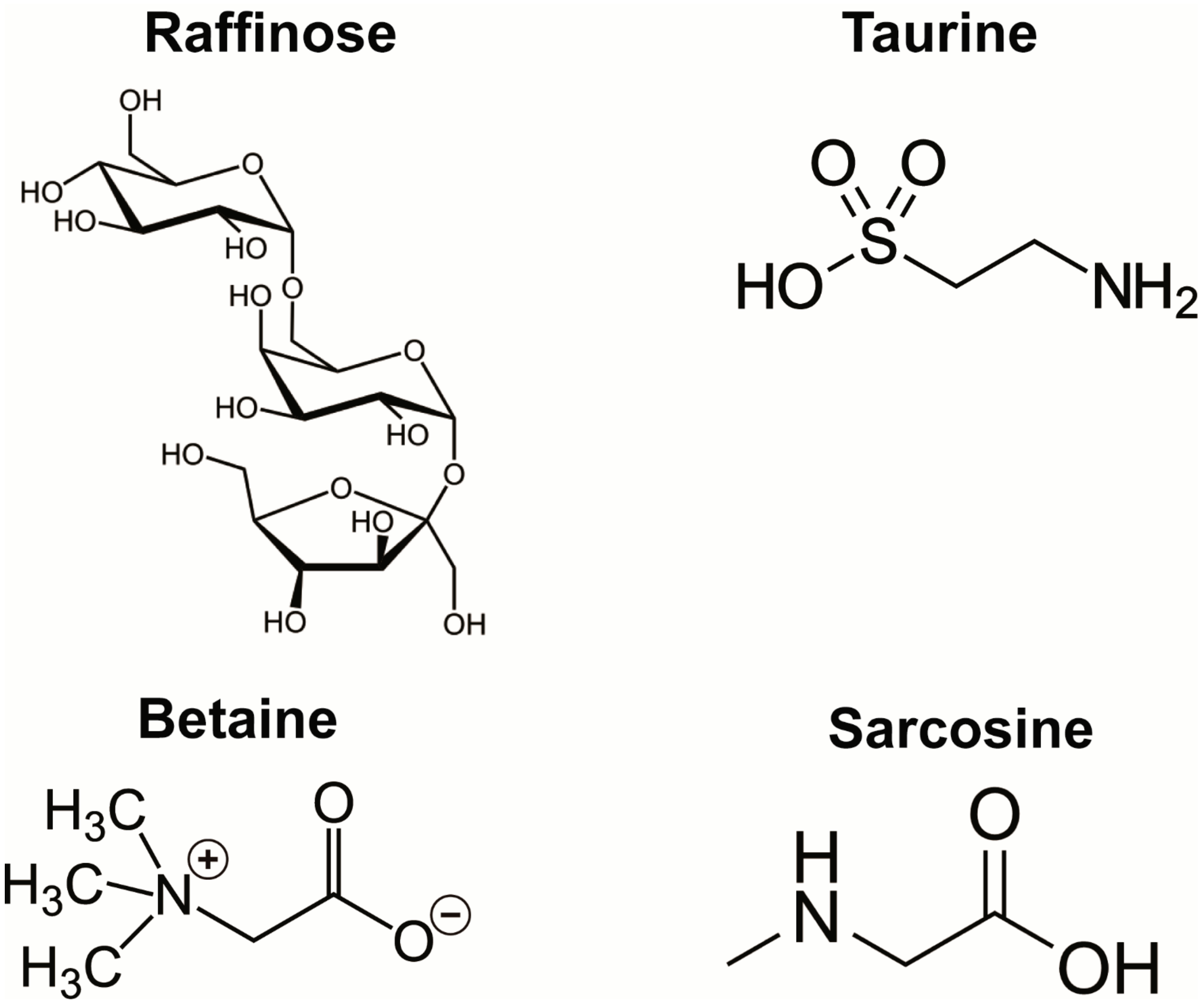

Supplementary Figure 2

Live cell imaging of the cultured fibroblasts: cells were incubated with varying concentrations of all four osmolytes (0.1 mM, 1 mM, 10 mM, 100 mM and 1000 mM) in triplicates. Images of cells were captured with IncuCyte ZOOM® System before and after addition of the osmolytes.

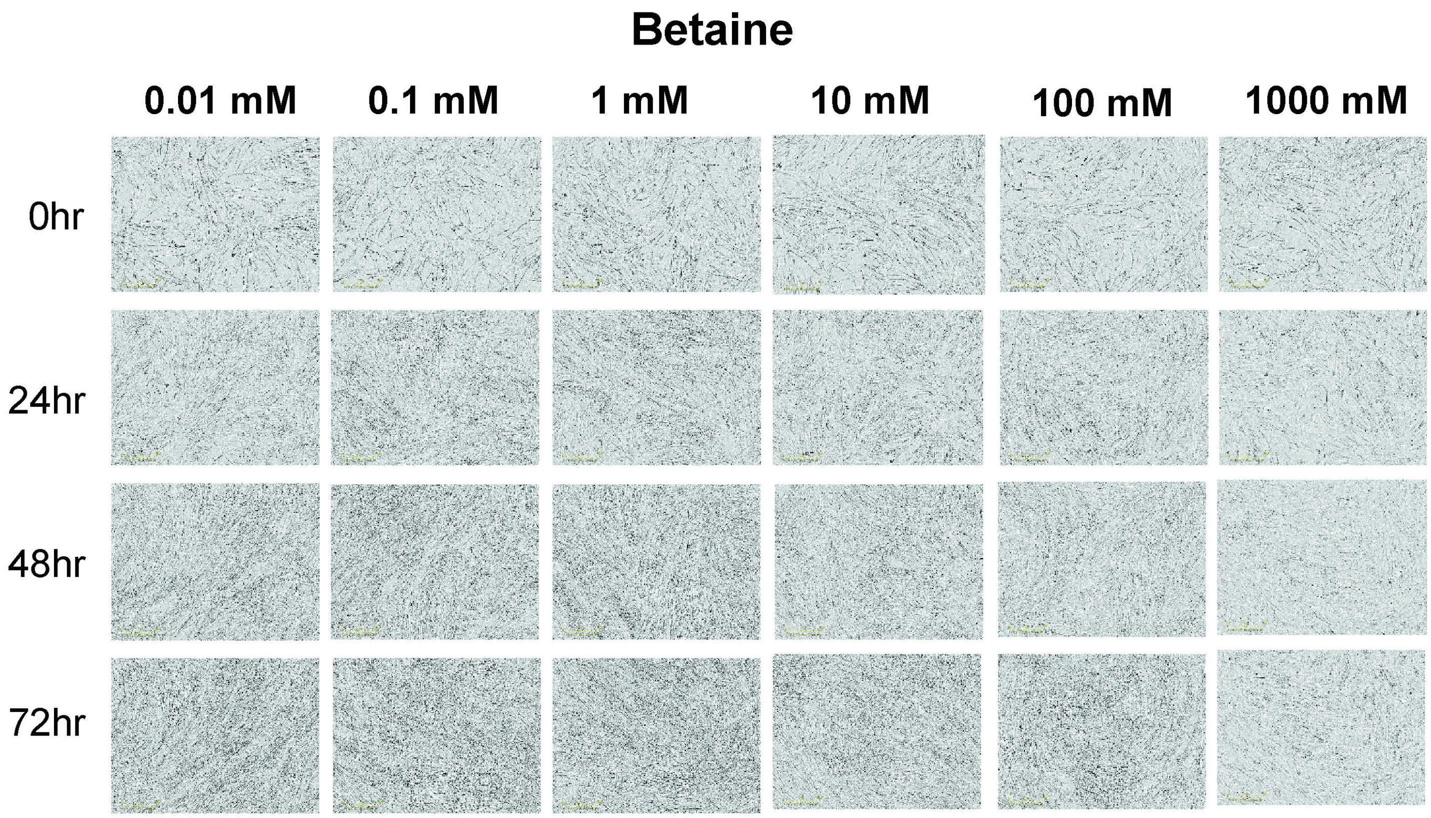

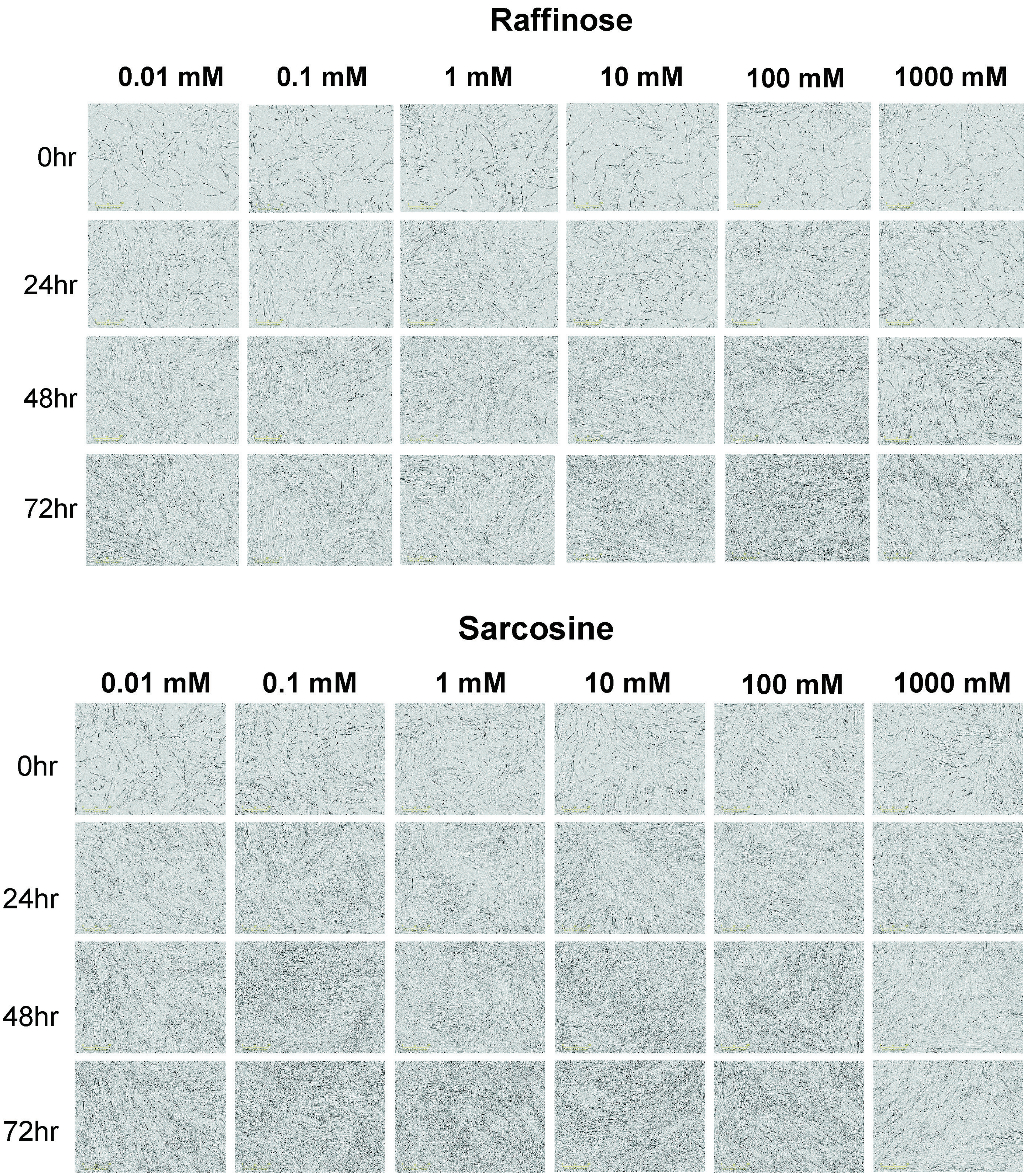
Raffinose
0.01 mM
0.1 mM
1 mM
10 mM
100 mM
1000 mM
0hr
24hr
48hr
72hr
Sarcosine
0.01 mM
0.1 mM
1 mM
10 mM
100 mM
1000 mM
0hr
24hr
48hr
72hr

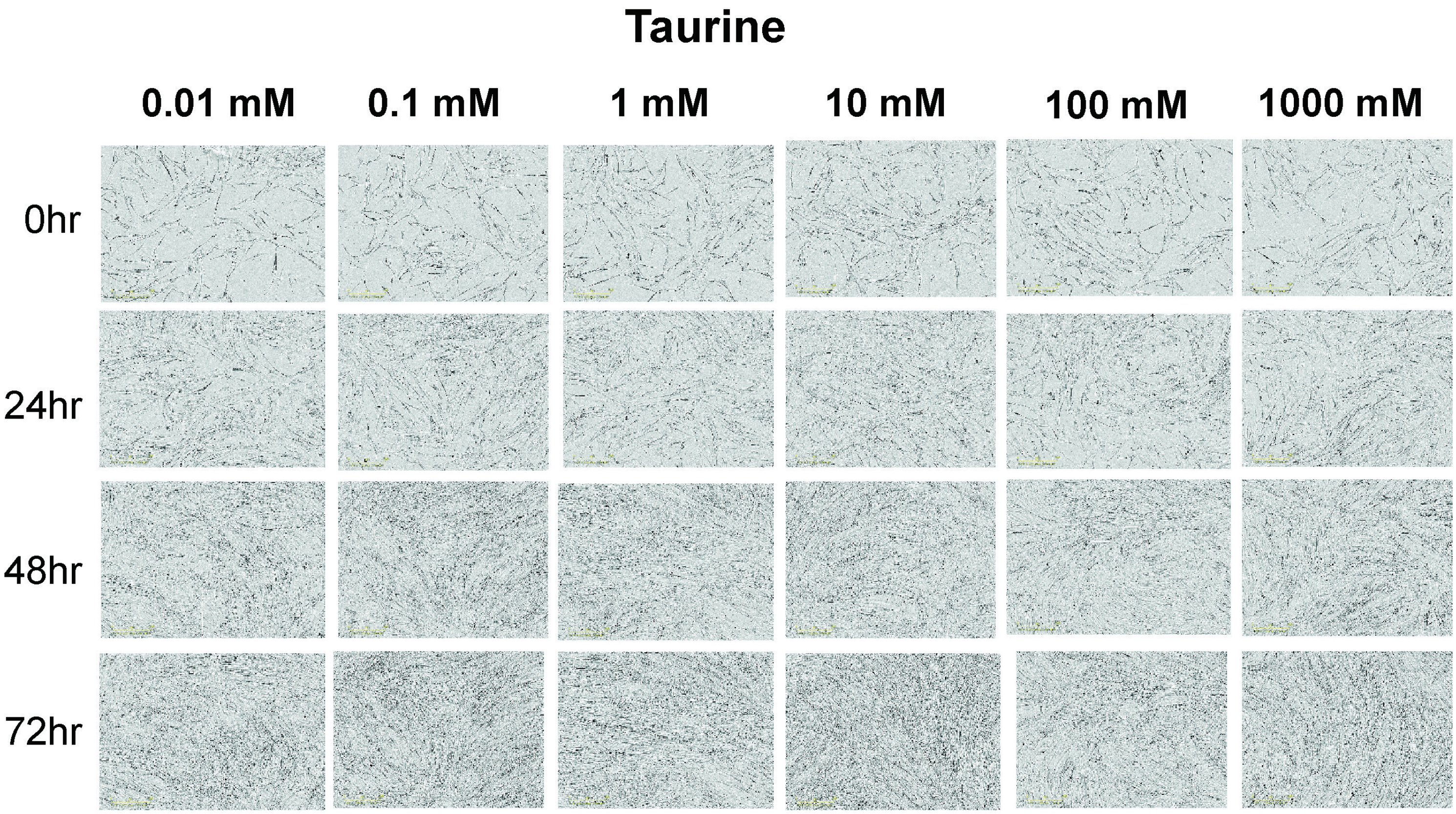
Taurine
0.01 mM
0.1 mM
1 mM
10 mM
100 mM
1000 mM
0hr
24hr
48hr
72hr

Supplementary Figure 2

Purity profiles of TGFBIp-G623R peptide by HPLC analysis after synthesis

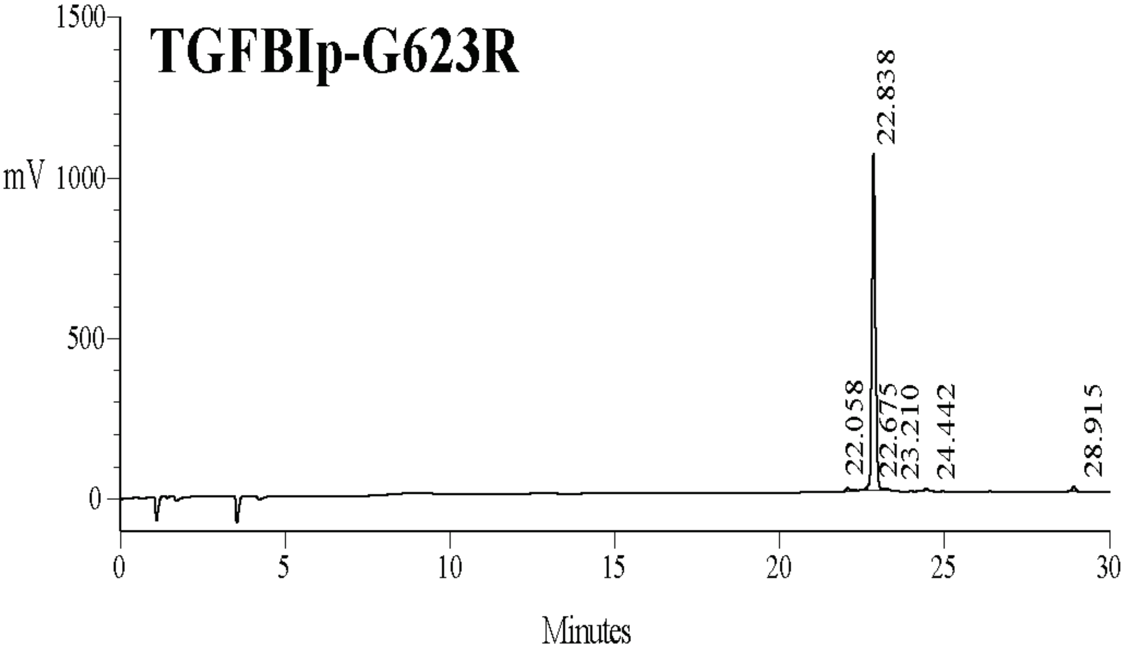

Table 1: Working concentrations of the different osmolytes used in this study

Osmolyte	Concentration (mM)	Molecular weight (g/mol)
Raffinose	200mM	504.4371
Taurine	200mM	125.1469
Sarcosine	200mM	89.0932
Betaine	200mM	117.1463

Supplementary Figure 3

Circular Dichroism spectra of G623R peptide at different time points

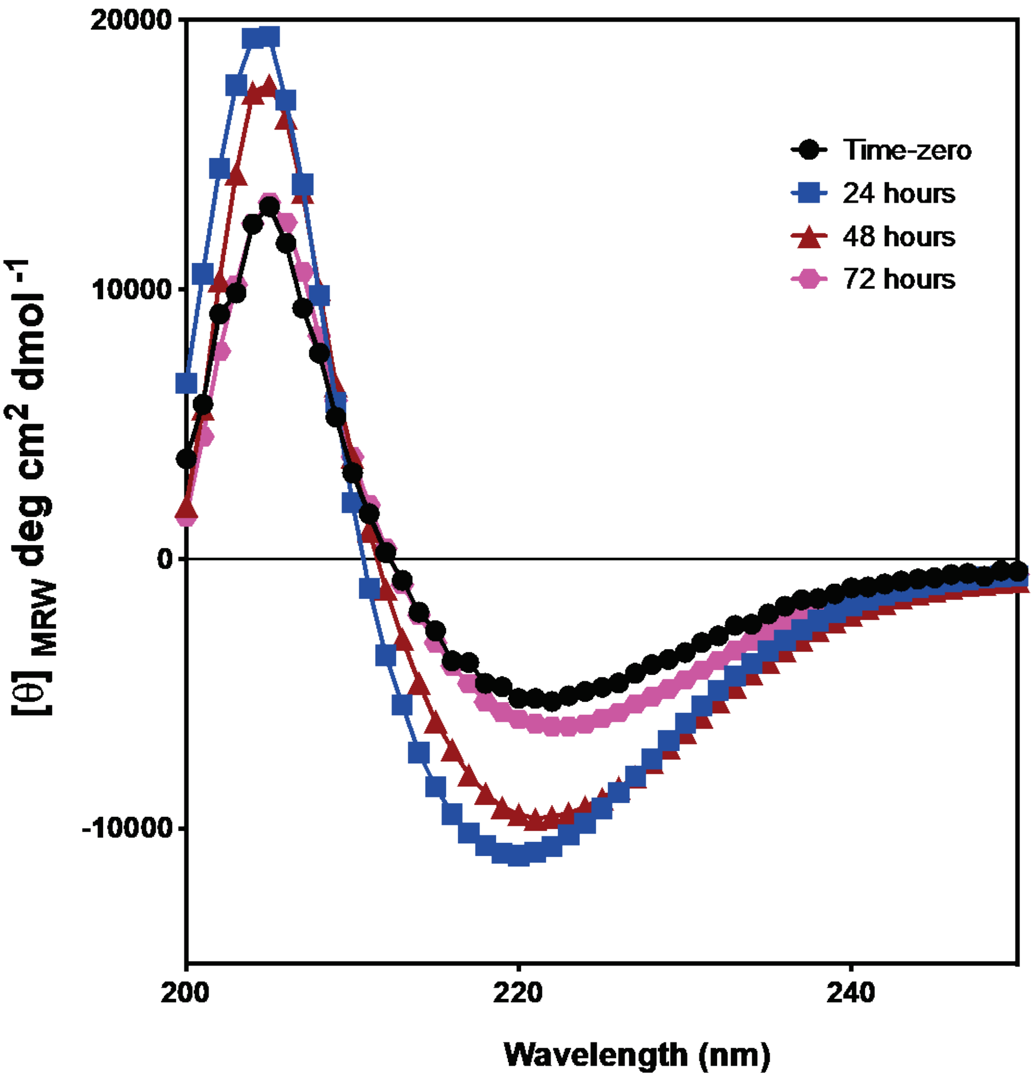

Supplementary Figure 4

Circular Dichroism spectra of G623R peptide at 2 different concentrations (0.6 mg/ml and 0.3 mg/ml) soon after it were dissolved in PBS.

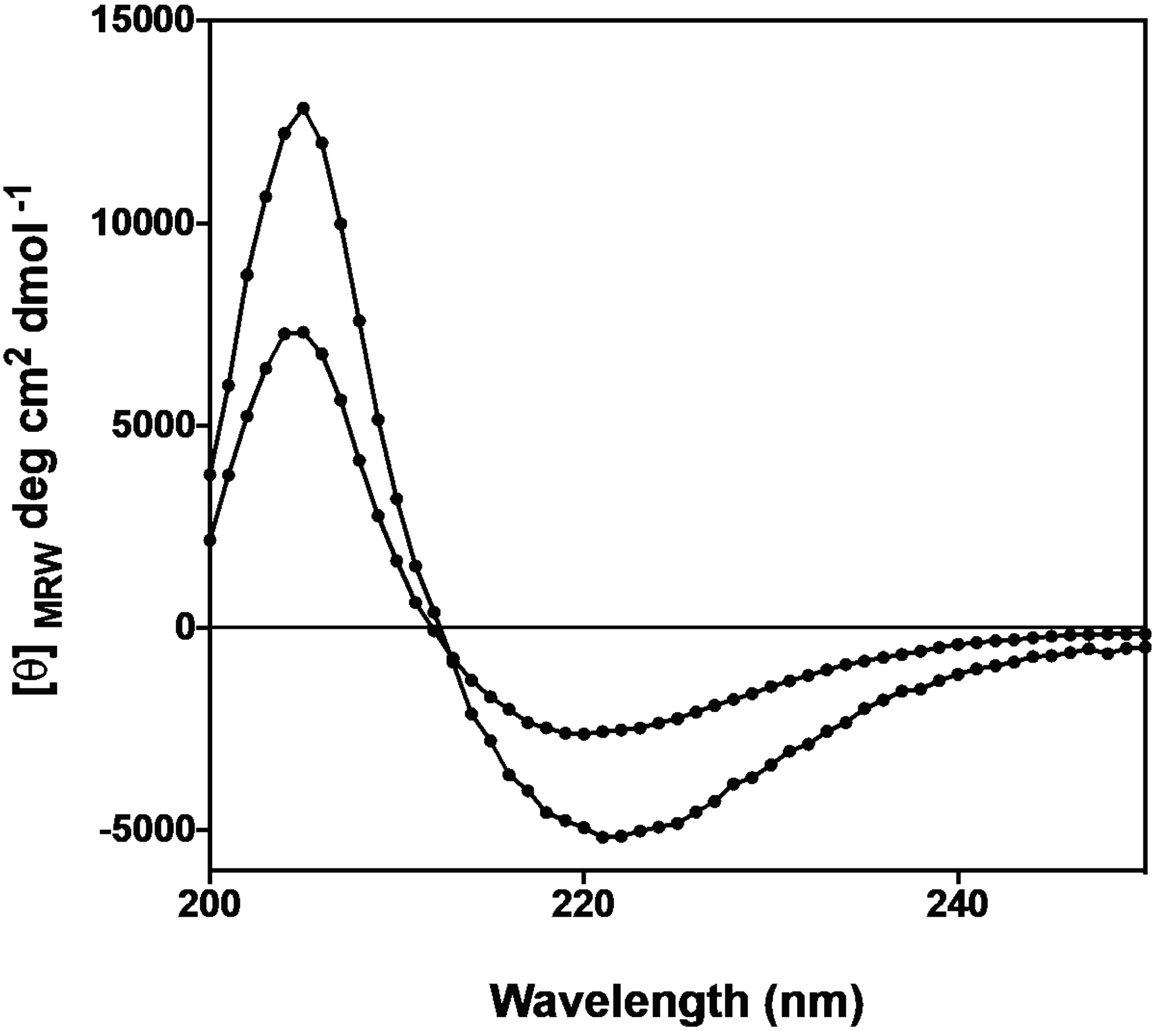

Supplement: Supplementary file 1 — Supplementary information. [file 41598_2020_60944_MOESM1_ESM.pdf]
